# Neurobiology of emotional regulation in cyberbullying victims

**DOI:** 10.3389/fpsyg.2025.1473807

**Published:** 2025-03-05

**Authors:** Sibin Mathew Nesin, Kriti Sharma, Kasturi Naresh Burghate, Madalaimuthu Anthony

**Affiliations:** School of Psychological Sciences, Christ University, Bangalore, India

**Keywords:** cyberbullying, neural pathway, emotional regulation, cyberbullying victimization, cyberaggression

## 1 Introduction

Cyberbullying is broadly defined as bullying that is carried out via electronic means such as text messages, emails, online chat rooms or social networking sites (Kowalski, 2013). In recent years, the increased use of social media and online communication increased the risk of cyberbullying. More than three billion people use social networks for communication (Chaffey, 2019). A recent study on 355 students in Jazan region, Saudi Arabia, aged between 12 and 18 years showed that ~20% of the participants spent nearly 12 h daily on the internet. The study showed that the prevalence of cyberbullying was 42.8% in male students, and 26.3% of the students' academic performance was affected by cyberbullying (Gohal et al., 2023). Another large sample study (*N* = 64,174) on school boys and girls revealed that 15% of the boys and 24.1% of the girls have reported being asked for personal information over the Internet. 9.1% of boys and 20.3% of girls have reported that they have been victims of cyberbullying, and 5.8% of boys and 13.2% of the population felt unsafe when in contact with someone over the internet. The study also revealed that the highest chance of becoming a victim of any kind of bullying in boys and girls is in grade 10 and grade 11 (age 16 ± 1; Salmon et al., 2018). A study carried out in around a thousand adolescents showed that 10.9% of the participants were involved in cyberbullying as bullies or victims (Carvalho and Branquinho, 2021). In the study, 8.5% of the participants were involved in both bullying and cyberbullying. The study also found that the bullies were using more tobacco and illicit drugs compared to victims.

Bullying has been increasingly studied recently because of its direct link to emotional distress, maladaptive social functioning, poorer health, and physical violence. Noteworthy has been the advent of cyberbullying in recent years, wherein the Internet and cellular phones are used to inflict harm on others intentionally (Reio, 2016). The psychological implications of cyberbullying are well-documented, spanning a wide range of negative impacts such as depression (Selkie et al., 2015), anxiety (Kowalski et al., 2014), low self-esteem (Extremera et al., 2018) and even suicidal thoughts (Extremera et al., 2018). However, the underlying neurobiological mechanisms that mediate these effects are less understood. Emerging evidence suggests that cyberbullying can lead to significant alterations in neural activity, particularly in brain regions associated with empathy, reward processing, emotional regulation, and self-referential thinking (McLoughlin et al., 2020). Functional magnetic resonance imaging (fMRI) research indicates that online social interactions are associated with similar structural correlates and patterns of brain activity to those observed in the context of real-world relationships (Lamblin et al., 2017). However, the number of neuropsychological studies addressing cognitive and emotional changes following cyber-victimization is surprisingly insufficient.

## 2 Psychological effects of cyberbullying

Victims of cyberbullying report significantly higher levels of anxiety and depression, and are express suicidal ideation (Kowalski et al., 2014) than victims of traditional bullying and cyberbullying has been found to have severe impacts on mental health (McLoughlin et al., 2020). In the present paper, traditional bullying involves face-to-face verbal or relational form of bullying that impact the mental health of victim. Emotions like feeling annoyed, fear for safety, frustration, and anger were present in the cyber-victims, along with sadness and anxiety (Hamm et al., 2015) and have a negative effect on victims' self-esteem (O'Brien, 2013). Young cyberbullying victims have reported sleep disturbance and bed-vetting (Monks et al., 2009). The cyberbullying victims also had lower levels of self-esteem (Palermiti et al., 2017).

The cyberbullying and psychological health are found significantly associated with each other. A negative association between self-esteem and a positive association between anxiety and depression were found. Psychological health (self-esteem, depression, and anxiety) of involved and not-involved respondents differ significantly (Baruah et al., 2017).

The victims of traditional bullying or cyberbullying had similarities in some of their behaviors (Keith, 2018). In the study, adolescent students of traditional bullying showed avoidance behavior (6.6%) and carrying weapons for self-protection (2.8%). Both traditional bullying and cyberbullying victims have shown an increase in the likelihood of fear of being harmed and expressed a correlation between fear and avoidance behavior but not between fear and weapon-carrying behavior. Low emotional clarity, low emotional regulation and low life satisfaction were observed in cyber-victims (Estévez and Cañas, 2020). An increase in the probability of showing loneliness, depression and stress was found in the victims, along with an increase in avoidance and social anxiety (Brandt et al., 2022). The traditional and cyber victimization was found to cause psychological maladjustments in the adolescent population (Eroglu and Peker, 2022). As many victims lack emotional clarity, they face difficulty in managing their stress and cognitive ability to take necessary actions, which eventually leads to maladjustment behaviors and unhealthy coping strategies (Estévez and Cañas, 2020).

The majority of the studies on cyberbullying were carried out in the adolescent population. Some of the strategies exhibited by the children and teenage cyber-victims include passive strategies like ignoring messages, blocking the sender and protecting personal information. Many victims also preferred active strategies like confronting the bully or fighting back. A smaller number of people preferred retaliation, and only 2% of the victims were found to engage in self-harm behavior following cyber victimization. A study has shown that childhood maltreatment has increased the risk of being a cyberbullying perpetrator later in their lives (Sun et al., 2020). Other studies also have revealed a positive correlation between childhood maltreatment and cyberbullying perpetration (Zhang et al., 2023). Aggression and anger rumination can later develop into bullying behavior. The maltreated children often express low self-control and express higher aggressive levels that may increase the possibility of expressing bullying behavior (Li et al., 2022). Understanding the neurobiology of emotional regulation in bullies can also help in developing strategies to provide support in reducing bullying behavior. Research on 524 Caucasian adolescents explored the empathy and emotional regulation of cyberbullies and victims (Arató et al., 2020). According to the study, cyber victims were more empathetic than the bullies, and those who were victims showed more significant concern for other victims, indicating that such social character could be an antecedent of cyber victimization (Nixon, 2014). The cyber-victims were found to use maladaptive emotion regulation strategies to manage in the early period following victimization and more adaptive strategies later (Schunk and Zeh, 2022). Maladaptive emotional regulation strategies include rumination, self-blame, acceptance and planning. Compared to the cyberbullies and outsiders (control group), the study reports that the victims showed more maladaptive emotional regulation (Arató et al., 2020). Victims experience a lack of acceptance in their peer groups, which results in loneliness and social isolation, low self-esteem, and depression; it can lead to stress-related disorders, concentration and school problems, emotional disorders, and even suicide (Roth, 2015).

To summarize, available evidence indicates that children who engage in cyberbullying in any form are more likely to experience psychological discomfort, including depression and anxiety symptoms, as well as worse subjective well-being.

## 3 Brain regions in emotional regulation

Emotional regulation includes the recognition of the emotional valance of stimuli, appreciating the need for emotional regulation, and implementing an appropriate strategy (Gross, 2014; Sheppes and Suri, 2015). The three major areas of emotional regulation are cortex, striatum and limbic system. Major cortical regions involved in emotional regulation are PFC, ACC and Insula. The limbic circuit involves experiencing, eliciting, learning and memory associated with emotional stimuli. The ventral striatum participates in reward and motivation associated with an emotional event and integrates emotion and cognition. Long-range amygdala connections like the hypothalamic-pituitary-adrenal (HPA) axis mediate animal stress response. Major subnuclei of the amygdala include the basolateral amygdala (BLA), central amygdala (CeA), and medial amygdala (MeA). CeA and BLA have a bidirectional connection with PFC, especially with mPFC and OFC, and play a role in the modulation of memory and learning and controlling goal-directed behavior.

The ventral striatum consists of nucleus accumbens (NAc) medial and ventral portions of caudate and putamen. The rostral part of NAc is responsible for “liking” an event/stimuli, and the caudal part signals “not-liking.” Amygdala (BLA), hippocampus and OFC send glutamatergic signals in synchrony to NAc responsible for “wanting,” valuing, and formation of memory associated with stimuli. NAc receives dopaminergic connections from the ventral tegmental area (VTA). vlPFC also sends glutamatergic inputs to NAc, and NAc has a strong connection to the insula. Elevated activity of anterior insula (AI) and dACC was found to be associated with high anxiety, risk avoidance, pain perception and neuroticism. Hyperactivation of AI, primarily ventral anterior insula (vAI), is associated with re-experiencing traumatic memories (Nicholson et al., 2020) and lateral PFC and dorsal ACC (anterior mid-cingulate cortex) dominate emotional regulation.

In contrast, the orbitofrontal cortex (OFC) and ventromedial prefrontal cortex (vmPFC) participate in decision-making and reward-related behavior concerning emotional stimuli (Rolls and Cheng, 2020). Some authors refer to the medial prefrontal cortex (mPFC) and orbitofrontal cortex (OFC) together as the ventromedial prefrontal cortex (vmPFC). The dorsolateral prefrontal cortex (dlPFC) is critical in cognitive control and executive functions, whereas the ventromedial prefrontal cortex (vmPFC) mainly involves emotional processing and reward-related decision-making (Nejati and Salehinejad, 2018).

The anterior cingulate (ACC) is a critical player in the corticolimbic system. The subgenual anterior cingulate cortex (sgACC) is an autonomic control center that controls the viscero-motor signals that modulate physiological responses like heart rate, blood pressure and neuroendocrine process. sgACC connects with regions like periaquiductal gray (PAG), hypothalamus, insula, amygdala, and OFC. sgACC helps regulate the PAG and hypothalamus concerning an emotional stimulus, and this appraisal exerts a top-down control on physiological response. The pregenual anterior cingulate cortex (pgACC) is found to be involved in subjective feeling to an emotional stimulus. The interconnection between pgACC and sgACC helps to function in the appraisal of positive and negative effects. pgACC is strongly activated when attention is directed internally (top-down) rather than externally (bottom-up).

Two major emotional regulation strategies are expressive suppression (ES) and cognitive reappraisal (CR; Gross, 2003). The CR strategy was shown to be more beneficial in managing emotions, while the ES strategy might lead to depressive symptoms (Goldin et al., 2008; Gross, 2003). The fMRI studies showed that major regions involved in ES are the ventrolateral prefrontal cortex (vlPFC), inferior frontal gyrus (IFG), insula and amygdala (Goldin et al., 2008). During CR, the recruitment of the dorsomedial PFC (dmPFC), dorsolateral PFC (dlPFC), vlPFC, insula, temporal cortex, parietal cortex and amygdala was found (Diekhof et al., 2011; Kalisch, 2009). Expressive suppression (ES) score was found to be high for males than females (Wang et al., 2017), and there was a positive correlation between ES and thickness of the superior frontal gyrus in males and a negative correlation in females. The study found significantly enhanced connectivity between the superior frontal gyrus and DMN regions in men during ES (Wang et al., 2017). A study by Hermann et al. (2014) found that ES was related to dorsal anterior cingulate/paracingulate cortex and medial PFC gray matter volume, where CR was positively associated with right and, tendentially, left amygdala volume. The dACC volume was positively correlated to CR but unrelated to ES (Giuliani and Drabant, 2011). Region of interest (ROI) and voxel-based morphometry (VBM) analysis found that the ES and not CR were positively related to anterior insula volume (Giuliani et al., 2011).

## 4 Adolescent brain and emotional regulation

The adolescent brain is under development. The synaptic pruning and myelination continue till early adulthood (Spear, 2013). The neural network for emotional regulation is still developing, leading to heightened social and emotional responses in the brain and behavior (Martin, 2016). Cortical thickness was found to vary from adolescent to adult. The white matter thickness is high, and the gray matter thickness is low compared to adults in the frontal and parietal cortices (Menary et al., 2013). The white matter increases till middle adolescence and then decreases until it stabilizes in early adulthood. The white matter thickness is an important feature to be considered as it reflects the inter and intra-hemispheric interactions (Dubois et al., 2014). The connection of the Prefrontal cortex (PFC) to other brain regions, like association areas and deeper brain structures, is necessary to develop social brain networks. Risk-taking behavior was found to be influenced both positively and negatively by social interaction during adolescence (Sakurai et al., 2015). Adolescence is critical for developing adaptive emotional regulation (Casey, 2013). Adolescents react to peer rejection with hypersensitivity compared to children or adults (Kloep, 1999). Emotional hyperreactivity, risk-taking behavior, instability in emotions, and challenges in social decision-making were higher in adolescence. Ostracism affects adolescent groups more as they place a high value on social rewards (Reyna, 2006; Steinberg, 2008).

The development of the limbic system happens early to the maturation of the prefrontal cortex (PFC). An MRI study on the teenage brain indicated the volumetric growth of the amygdala, and NAc was not associated with ES or CR (Guadagno et al., 2018). The study is promising as it suggests that the emotional regulation network in the adolescent brain is not yet developed fully, and there is a chance of developing intervention strategies that can help bully-victims (Ferschmann et al., 2021). Any emotional trauma during adolescence also affects the development of the emotional and social networks that could impact the person's adulthood (Downey, 2022). This makes the adolescent population vulnerable to bullying and cyberbullying. Also, traumatic events can affect the normal development of these networks and thus, the importance of addressing the issue of cyber-bullying seriously (Chadwick, 2014).

Synaptic density is higher during adolescence than adulthood, and synaptic pruning continues from childhood to adulthood (Huttenlocher, 1987). Synaptic pruning is believed to play a major role in more efficient cognitive processing (Blakemore, 2008). In the “Triadic Systems Model” (Ernst and Hale, 2014), the imbalance between the three regions, i.e., prefrontal cortex, Striatum and amygdala, helps in regulatory control of approach behaviors (rewarding stimuli) and avoidance behaviors (aversive stimuli). Many animal and human studies have shown heightened amygdala activity in response to emotional stimuli (McRae et al., 2012; Pfeifer et al., 2011). The elevated functioning of the ventral striatum is associated with less risk-taking behavior and increased resistance to ostracism (Masten et al., 2009; Pfeifer et al., 2011). Diminished activity of the ventral striatum was found to correlate with increased depressive feelings in adolescent individuals. The three regions, the Prefrontal cortex (PFC), amygdala and ventral striatum development, are not balanced. The amygdala develops earlier than PFC, and this developmental mismatch in adolescence is prominent, leading to emotional dysregulation (Somerville and Jones, 2010; Steinberg, 2008).

## 5 Impact of bullying and cyberbullying on emotional regulation

A study by Erik de Water showed that social exclusion caused the activation of the dorsal anterior cingulate cortex (dACC) and was positively linked to participants' acceptance, where the victims exhibited increased activation of the medial prefrontal cortex (mPFC; de Water et al., 2017). Another study (Telzer et al., 2021) showed that adolescent girls with a past of severe victimization had heightened activation in neural regions associated with emotional processing, mentalising, and social perception, which can lead to increased social monitoring. High peer verbal abuse in adolescence showed a high depressive score with increased activity in the left ventrolateral prefrontal cortex (VLPFC) and higher connectivity between the left VLPFC and left hippocampus (Lee et al., 2017). The victims during exclusion showed higher activation in social pain, dorsal anterior cingulate cortex (dACC), subgenual anterior cingulate cortex (sgACC) and high internalizing symptoms as compared to the participants who were not the victims (Rudolph et al., 2016). Ostration displayed higher dACC activation while experiencing rejection and higher dorsal anterior cingulate cortex (dACC) and anterior prefrontal cortex (aPFC) activation when they were incidentally excluded from social interactions where they were involved. Perceiving low relational value during fMRI changes in frontostriatal connectivity. Social exclusion was found to trigger the activity in areas of the frontal brain, which are sub- and pregenual ACC, dorsolateral PFC, and left interior frontal cortex (Grosshagauer et al., 2024). It has been observed that there was a correlation between the increase in exclusion-focused brain activity and various past experiences with bullying, especially in left IFG and sgACC (Kiefer et al., 2021). Studies showed greater activation in the amygdala and inferior fusiform gyrus during social exclusion (McIver et al., 2019; Rudolph et al., 2016) reported that the two brain regions ACC and insula are associated with heightened neural activity in response to social exclusion compared to inclusion. This heightened activity shows a positive correlation with self-reported distress. Schriber et al. (2018) showed that excluded adolescents were affected by past hostile school environment exposure and found that subgenual anterior cingulate cortex (subACC) responses during the exclusion event have forecasted increases in adolescent depressive symptoms and experiences with social and physical pain. Using a combination of neuroimaging techniques and self-report measures (Oppenheimer et al., 2020), found the extent to which altered neural processing of social rejection in two key brain regions, insula (AI) and dorsal anterior cingulate cortex (dACC), interacted with negative social experiences to predict suicidal ideation.

Limited research is available on neural correlates of cyber victimization, and the field has yet to receive scientific attention. A piolet Blood-oxygenation-level-dependent (BOLD) task-based fMRI (tb-fMRI) study by McLoughlin et al. (2020) was conducted on 32 young adults age ranging from 18 to 25 years old. Females, compared to males, had greater activation of the right ACC while observing cyberbullying stimuli as compared to neutral stimuli. The caudo-dorsal ACC (area 24) is also involved in cognitive control network (CNN), performance monitoring and error detection. The activation of the left and right middle temporal gyrus was found to be significantly higher in cyberbullying stimulus as compared to neutral stimuli, indicating the influence of cyberbullying on social cognition and emotion. The study also showed increased recruitment of the left angular gyrus (Brodmann area 39) in the posterior part of the inferior parietal lobule. This region is actively involved in DMN (Cubillo, 2022).

Another study on cybervictims by McLoughlin et al. (2022) showed that the cerebellum's left dorsal caudate and crux I showed higher activation in males (aged 18–25 years) than cerebellum's left dorsal caudate and crux I showed higher activation in males than females. In contrast, the right rostroventral area of the cingulate gyrus showed activation in both males and females. The crux I of the cerebellum and dorsal caudate is involved in cognitive aspects of emotional processing (Adamaszek et al., 2017).

A study by Morese et al. (2024) explored the emotional response of cyberbullying victims to virtual social exclusion and inclusion through a cyberball task. The study was conducted on the adolescent population, and data showed that a high level of empathy was associated with the experiences of social exclusion in the cyberball paradigm. González-Cabrera et al. (2017) found that cyber-victims had higher cortisol secretion and more significant perceived stress in adolescent age groups, and a study by du Plessis et al. (2018) on traditional bullying showed that in boys the victimization was correlated with high cortisol levels and smaller vlPFC.

## 6 Conclusion

Evidence-based literature is scarce on the effect of cyberbullying on the brain. Neuroimaging and electrophysiological studies should be promoted to understand the underpinnings of emotional dysregulation in the adolescent population following cyberbullying. Different emotional regulation strategies involve various regions of the brain, and the cognitive flexibility in adapting the positive strategies needs to be studied in detail. Victims with high resilience adapt positive emotional regulation strategies compared to those with emotional dysregulation. Conducting more neuropsychological studies to explore the underlying mechanism can lead to developing new rehabilitation strategies to help the vulnerable population.
